# Depersonalization disorder as a systematic downregulation of interoceptive signals

**DOI:** 10.1038/s41598-022-22277-y

**Published:** 2022-12-21

**Authors:** Fedal Saini, Sonia Ponzo, Francesco Silvestrin, Aikaterini Fotopoulou, Anthony S. David

**Affiliations:** 1 Institute of Psychiatry, Psychology and Neuroscience, King’s London College, London, SE5 8AF UK; 2 Flo Health, London, UK; 3 Institute of Health Informatics, University College London, London, UK; 4 Thrive Therapeutic Software Ltd., London, UK; 5 University of East Anglia, Norwich Research Park, Norwich, Norfolk NR4 7TJ UK; 6 Division of Psychology & Language Sciences, Clinical, Educational & Health Psychology Research Department, University College London, London, UK; 7 Institute of Mental Health, Faculty of Brain Sciences, University College London, London, UK

**Keywords:** Learning algorithms, Human behaviour

## Abstract

Depersonalisation disorder (DPD) is a psychopathological condition characterised by a feeling of detachment from one's own body and surrounding, and it is understood as emerging from the downregulation of interoceptive afferents. However, the precise mechanisms that drive this ‘interoceptive silencing’ are yet to be clarified. Here we present a computational and neurobiologically plausible model of DPD within the active inference framework. Specifically, we describe DPD as arising from disrupted interoceptive processing at higher levels of the cortical hierarchy where the interoceptive and exteroceptive streams are integrated. We simulated the behaviour of an agent subjected to a situation of high interoceptive activation despite the absence of a perceivable threat in the external environment. The simulation showed how a similar condition, if perceived as inescapable, would result in a downregulation of interoceptive signals, whilst leaving the exteroceptive ones unaffected. Such interoceptive silencing would force the agent to over-rely on exteroceptive information and would ultimately lead to the DPD phenomenology. Finally, our simulation shows that repeated exposure to similar situations over time will lead the agent to increasingly disengage from bodily responses even in the face of a less triggering situation, explaining how a single episode of depersonalization can lead to chronic DPD.

## Introduction

Depersonalisation disorder (DPD) is a psychopathological condition characterised by a persistent and distressing alteration in the quality of a person's subjective experience of themselves (depersonalisation), which can be accompanied by a modified perception of one's surroundings (derealisation). DPD symptomatology is mainly characterised by emotional numbing (i.e., “de-affectualisation"^1–3^), together with a feeling of detachment from one's own body^4^. Mild and transient DPD episodes are a common phenomenon, with a life prevalence estimated at 74%, and often results from stress and fatigue^5^. More serious forms of DPD may be associated with a previous history of anxiety and panic disorder^6–8^, and symptoms of depersonalisation frequently accompany psychiatric conditions such as post-traumatic stress disorder, schizophrenia, panic disorder and depression^5,9^. Despite the vivid nature of such feelings of detachment, patients' ability to distinguish between subjective and objective reality remains intact.

Several attempts to explain the aetiology of DPD have been made in recent years^10^. One model, developed by Sierra and David^11^, suggests that DPD may arise as a consequence of an increased cognitive control of the subjective affective experience. This idea is based on the observation of a reduction of anterior insula (AI) activation in response to emotional stimuli, together with increased lateral prefrontal activation in DPD patients as compared to healthy controls^12,13^. The insula is a cortical area receiving information about the internal state of the body and it is considered a key region of emotional and bodily awareness processing^14^. Conversely, lateral prefrontal cortices are largely involved in emotion and action regulation^15–17^, inhibitory control^18,19^, as well as goal-appropriate response selection^20,21^ and are thought to exert inhibitory control over the insula. As put forward in Sierra and David's model, in DPD lateral prefrontal cortices employ an excessive inhibitory control over the insula, dampening the emotional experience and giving rise to a subjective “feeling of unreality"^11^ (p. 99). Accordingly, DPD patients exhibit autonomic responses to negative emotional stimuli that are blunted compared to those of healthy controls^22^, and inversely related to lateral prefrontal activation^23^, thus supporting the hypothesis of an inhibitory role carried out by frontal areas. This fronto-insular inhibitory mechanism has also been observed in healthy individuals during voluntary negative affect suppression tasks, thus suggesting that the emotional detachment manifested in DPD may be the result of a pathological enhancement of an otherwise healthy control mechanism^24^. Two case studies^25,26^ and two trials^27,28^ demonstrated the directionality and causality of the fronto-insular inhibitory circuit by reporting temporary reduction in DPD symptoms immediately after the delivery of inhibitory magnetic stimulation over the lateral prefrontal cortex. Interestingly, excitatory magnetic stimulation over the same prefrontal areas has been shown to give rise to DPD symptoms in a treatment-resistant depressed patient^29^.

One potential candidate explanation for such emotional detachment is that, in DPD patients, information relating to incoming interoceptive signals is suppressed. Interoception, the sense of the state of one's own body^14^, plays an important role in emotion regulation^30,31^, social ability^32–34^, motivation^35,36^, decision making^37–43^, attachment^33^, and self-monitoring of arousal^43^, hunger^44^ and pain^45^ and hence a tangible sense of self. Given its crucial role in several aspects of mental and physical health, its disruption has been associated with several psychiatric disorders, including depersonalisation (see^46^ for a review).

The aim of the current paper is to provide a computational and neurobiological model of depersonalisation as arising from disrupted interoceptive processing at higher levels of the cortical hierarchy. We will start by describing the theoretical framework of reference, Predictive Coding and Active Inference under the Free Energy Principle. We will then review relevant literature investigating Predictive Coding accounts of interoception and their role in DPD. Finally, we will outline the generative model underlying our proposed candidate mechanism causing DPD and illustrate such mechanism via simulation of an agent’s behaviour in a DPD episode.

### Predictive coding and active inference

A theoretical framework that has proven useful in outlining potential disruptions in interoceptive processing in DPD is that of Predictive Coding (PC) and Active Inference under the Free Energy Principle^47,48^. The core idea behind this account is that the brain acts as a Bayesian inference machine (a concept shared by other probabilistic accounts of brain function, e.g.,^49–51^). Biological agents do not have direct access to the states of the outside world, or even of their own organism, but must infer these (hidden) states by combining noisy sensory evidence (hereinafter referred to as observations) with predictions, following the Bayes rule^52^. To make predictions, one must have some structural knowledge of the environment, or, in other words, an internal model of it. We call these *generative models* because they specify (in a probabilistic manner) how hidden states generate observations. These models have two types of unknowns: time-varying, situation-specific latent variables (i.e., the aforementioned hidden states) and more slowly varying (if at all), generalisable model parameters. We call the process of deriving the value of hidden states from observations *inference* and that of updating model parameters *learning.* Thus, every time an agent encounters a stimulus (whatever its modality), it must infer its causes (hidden states) by combining the observation itself and prior knowledge and update its internal model to make better predictions in the future. This happens at all levels of the processing hierarchy, and the higher the hierarchical level, the more information originating from different streams will be integrated. In this framework, perception is nothing but inference performed at low hierarchical levels^52^.

A popular implementation of this idea is the Free Energy Principle (FEP)^53^, which frames all brain activity as an attempt to maximise a quantity known as variational free energy (VFE; Note that in the FEP literature the sign of VFE is often reversed, and authors often refer to VFE minimisation. We chose to keep the sign as in the machine learning literature^54^. This means the brain would perform a certain type of approximate Bayesian inference, called variational inference(see^50^ for a discussion of why the brain cannot perform exact inference and has to resort to approximations). We won’t discuss the FEP in the detail here (see^53^ for a discussion, and see^55^ for a critical overview). For our purposes, it suffices to say that minimising VFE is equivalent to minimising surprise in the long term (i.e., adjusting one’s internal models to better account for both present and future observations). PC is an algorithmic implementation of this principle, with some assumptions in place, the most important being a generative model with Gaussian form^56,57^. In this framework, inference can be seen as the interplay of top down predictions and bottom up prediction errors^56,57^, the core goal of the brain would be to adjust predictions so that they can effectively suppress (or “explain away”) prediction errors at all levels of the cognitive hierarchy. It can be shown that, once a generative model with Guassian form is assumed, maximising VFE is indeed equivalent to minimising prediction error.

Neurobiologically realistic implementations of PC (e.g.,^58,59^) model this as an interplay between "representation units", encoding the value of a certain variable the brain is trying to infer, and "error units", representing the variance-weighted difference between top-down predictions and bottom-up signal (i.e. prediction error; note that some work in this area does not refer to variance, but rather to its inverse, precision). Signal variance is a crucial quantity in PC, as it regulates the relative weight of different information sources in information integration. This holds both for information coming from different channels (e.g., “how much do I trust visual versus auditory information?”) and from different hierarchical levels (e.g., “how much do I trust my priors versus sensory evidence?”). Furthermore, signal variance has been suggested to be involved in many psychiatric disorders^60,61^, and in this paper we will argue it may play a central role in DPD as well.

**Figure 1:**
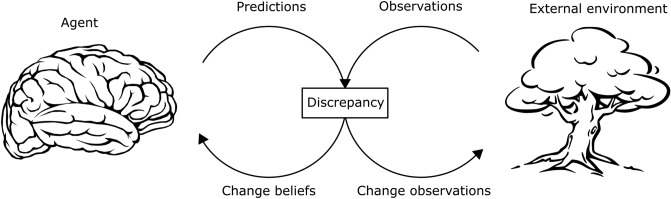
Schematic illustration of PC and Active Inference. As an agent (left) interacts with an external environment (right), it will make predictions about the stimuli it will encounter. In most cases, these predictions will not perfectly correspond with the incoming observations, and there will therefore be a discrepancy between the two (also called prediction error). The agent can reduce this discrepancy by changing its beliefs, or alternatively, by acting upon its environment, changing its own observations to fit its predictions.

In standard PC accounts^56^, predictions about sensory observations represent beliefs about hidden states. Once an observation is encountered, predictions will initially correspond to priors (i.e., beliefs prior to stimulus exposure). Inference involves updating such beliefs, until the best compromise between priors and sensory evidence (possibly coming from different channels) is reached. However, one does not necessarily update beliefs to make them match sensory observations. Many biological agents (including, of course, humans) can change their own observations by acting upon their environment. They can, in other words, modify their observations to make them match their predictions, instead of the opposite. Within the FEP, this idea takes the name of Active Inference^57^. Here priors over hidden states are conceptualised as preferences or goals^47^, and while some of these might be susceptible to change (e.g. circumstantial goals), the ones linked to survival are likely to be hardwired (e.g. the preference of being safe versus in danger, full versus starving). In either case, in this conceptualization achieving goals (or preferred states) is equivalent to modifying sensory observations through actions to fulfil one’s predictions (see Fig. 1). Priors over actions (hereinafter referred to as policies) represent habits, and their parameters can be updated over time (i.e., they are subject to learning), equivalently to priors over hidden states.

An example of this would be that of an individual encountering a dangerous animal. If one thinks of “level of danger” as a hidden state, it makes sense for any individual to prefer its value to be low. Its prior thus would have higher probability associated with low values and low probability associated with higher values. There is therefore a mismatch between predictions (i.e., “I am safe”) and sensory evidence (“there is a dangerous animal nearby”). There are two ways to resolve the mismatch: (a) to update beliefs (and simply accept to be in danger) or (b) to act to change sensory observations and make them match preferences (i.e., act to get out of danger by running away). Of the two options, the second is clearly preferable, both from a common-sense point of view and from a mathematical one. In fact, shifting beliefs so that they are at odds with priors would result in lower VFE, if we assume that priors (preferences) over being in danger are lower than the priors over running away (which is a reasonable assumption).

In practice, however, things can be more complicated than this. In the context of our example, how could an individual be certain they will be able to outrun the dangerous animal? In other words, what is the probability of a certain action having a certain effect? This adds a layer of complexity to Active Inference models, as these probabilities are themselves beliefs, which can be updated and can change with the context. In this paper, we consider an extreme case: we assume our simulated agent to assign a probability of zero to any change in the observations as a result of any action (which we will refer to as “policy”). In other words, the agent implicitly assumes that no matter what it does, it will be unable to change its observations, and its situation is thus perceived as inescapable. We then include dissociation as a policy that, despite not changing observations directly, has an impact on how they are processed by inflating the variance associated with them.

### Predictive coding and interoception in DPD

In recent years, PC accounts of interoception have been proposed^62–64^ which, following the same principle of PC in other sensory modalities, state that expectations about the internal state of the body are deployed in the form of top-down prediction signals that are meant to suppress (“explain away") interoceptive prediction errors. Such processes are thought to culminate in the anterior insula (AI) and, when successfully implemented, will be made available at the conscious level as affect or sense of presence.

In this context it has been proposed that DPD may arise from an excessive but undifferentiated suppression of interoceptive signals^63^. However, while this model provides a useful starting point, the mechanism underlying what seems to be a generic suppression of interoceptive processing in DPD remains to be explained. As frequently reported in the literature^8^, DPD symptoms can arise as a consequence of an intense experience, such as severe stress, panic attacks or drug use and this is more common among individuals with a history of high trait anxiety, panic attacks, and childhood trauma^65,66^. In a more recent predictive coding conceptualization of depersonalization and derealization, Gatus and colleagues^10^ suggested that these disorders may be the result of imprecise interoceptive predictions arising from traumatic experiences and leading to an over-weighting of other sensory modalities. An alternative explanation is the one put forward by Ciaunica and colleagues^67^. They described DPD as arising from the failure in “somatosensory attenuation” (i.e., the phenomenon by which self-generated sensations are processed “transparently” in the background), which leads to detachment of the self. Within this account, the precision weighting appears systematically imbalanced towards self-priors, failing to flexibly update the internal model when new information is obtained.

In line with the interoceptive suppression hypothesis, we propose that depersonalisation is the result of an attempt to cope with a situation characterised by abnormally high physiological activation (as in the aforementioned conditions) and perceived as inescapable. We outline a candidate mechanism both at a computational and neurobiological level, arguing that prefrontal suppression of interoceptive prediction error units in the AI can result in preventing interoceptive signals from being processed at higher levels of the cortical hierarchy, ultimately leading to a blunted, disembodied perception of the self (“interoceptive silencing”). It has to be noted that we are not suggesting a suppression of interoceptive signals at the level of the posterior insula, but rather that this silencing mechanism takes place at a higher level of the hierarchy (AI).

We illustrate how this can occur in a simulation using an active inference algorithm, showing that, in the perceived absence of alternatives, the simulated agent will disengage from its abnormal interoceptive signals. We also show how, if such a situation presents itself frequently, the agent will update its habits accordingly, making depersonalisation episodes easier to trigger and longer in time. We are remaining agnostic about possible mechanisms that might contribute to developing an abnormal physiological activation (although see^68^ for an active inference account of this) and use that situation as our starting point.

## Results

Here we report the results of our simulation. Refer to the “Methods” section for the mathematical notation.

### Habits formation

**Figure 2:**
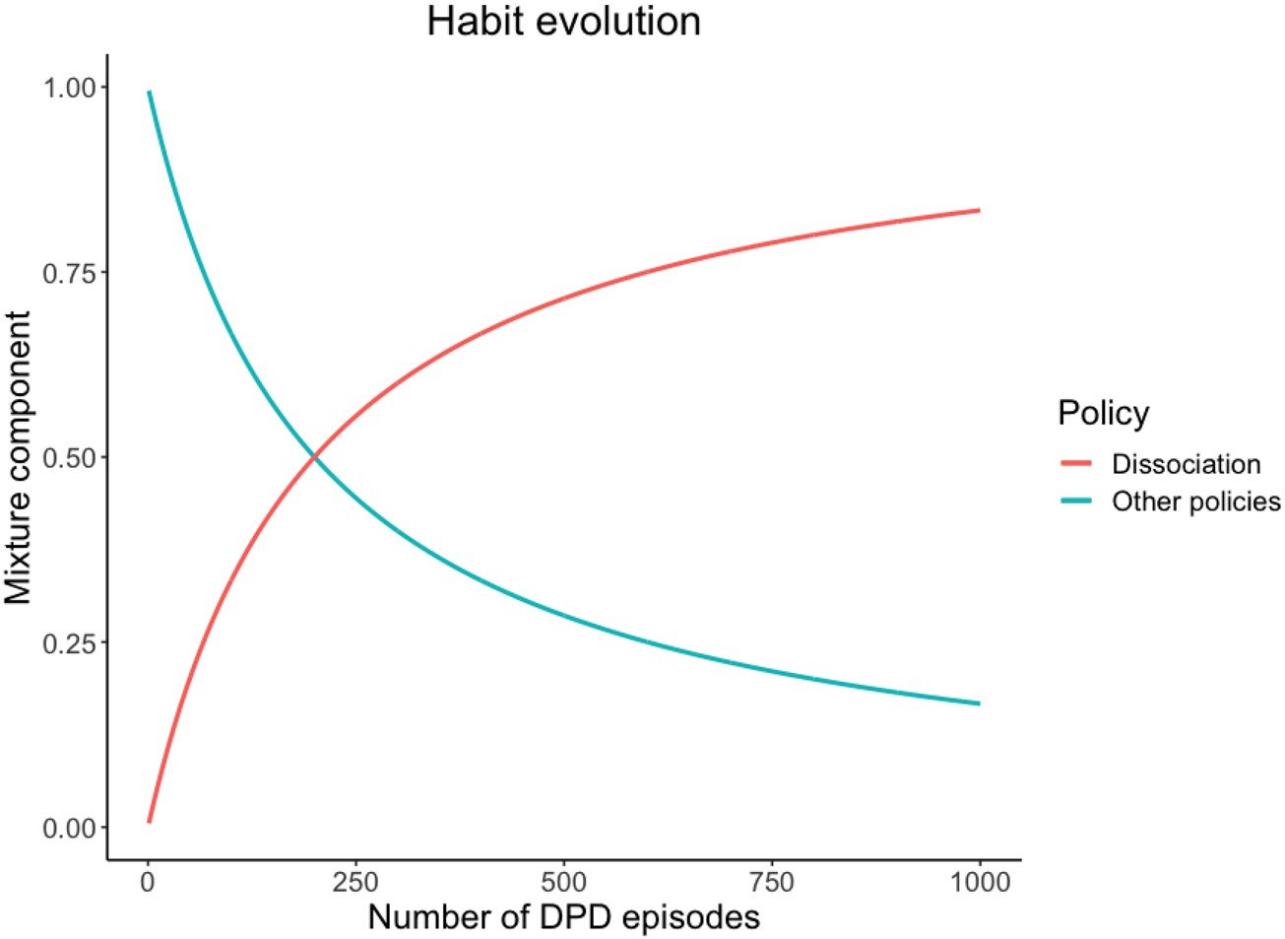
Evolution over time of the prior probability of enacting dissociative policies, regardless of observations. For purely illustrative purposes, here we show the effect of 1000 consecutive DPD episodes.

We first had our agent experience conflicting interoceptive and exteroceptive observations to simulate the development of depersonalisation habits. In other words, by feeding it abnormally high interoceptive observations (signalling danger) and non-threatening exteroceptive ones (signalling safety), we forced repeated depersonalisation episodes on our agent, which in turn brought it to assign an increasingly high prior probability ${\pi }_{d}$ to dissociative policies (see Fig. 2).

### DPD episode

**Figure 3:**
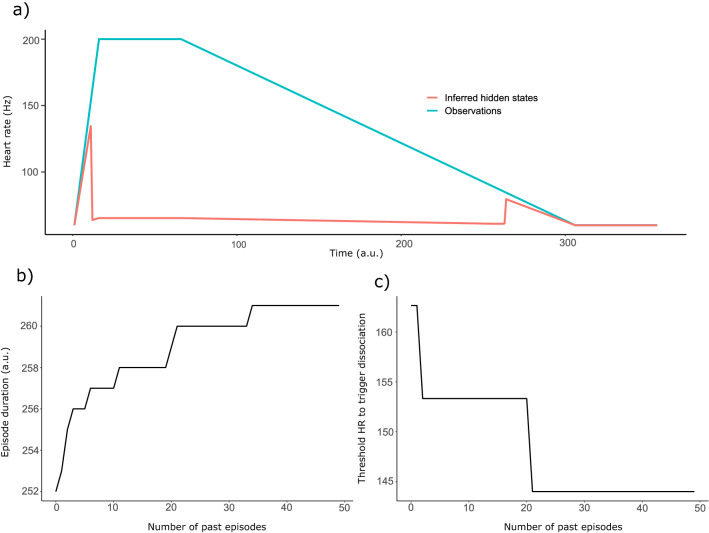
We plotted our results transforming all values to resemble realistic heart rates (HR) for illustration purposes. (**a**) Simulation of a DPD episode, with inferences about interoceptive lower-level hidden states (HR in this case) plotted in red and observations (i.e., actual HR) plotted in blue. We arbitrarily choose the first DPD episode in the agent’s lifetime to illustrate interoceptive silencing. As heart rate starts rising quickly, the agent disengages from it, cutting it out from higher level inferences (‘interoceptive silencing’). (**b**) Episode duration plotted as a function of the number of past DPD episodes the agent has experienced. The more the agent is used to dissociate, the longer it will dissociate for. (**c**) Minimum HR required to trigger a DPD episode plotted as a function of the number of past DPD episodes the agent has experienced. As the agent experiences more and more episodes, the easier it is to trigger a new one.

We then introduced a temporal component to the simulation, with interoceptive observations quickly rising to abnormal levels (coinciding with ${{\varvec{\upmu}}}_{2}$, signalling an unwanted higher-lever state), plateauing and then slowly returning to normal, while exteroceptive observations stayed stable at non-alarming levels (coinciding with ${{\varvec{\upmu}}}_{1}$). Actions played out proportionally to $\widetilde{\mathbf{c}}$, with ${\widetilde{c}}_{d}$ turning out to be always very close to 0 or very close to 1, displaying an on–off behaviour (see Fig. 3) that made policy sampling unnecessary.

**Figure 4:**
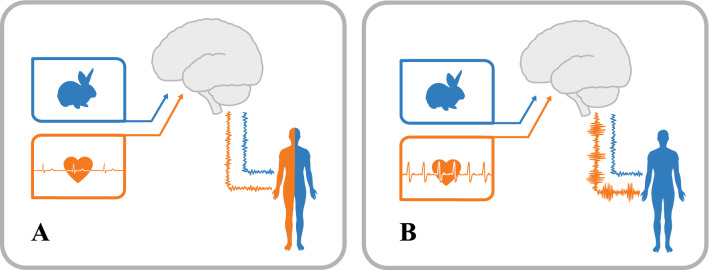
In (**A**), the interoceptive information is congruent with the exteroceptive ones (low heart rate when seeing a rabbit). None of those information streams are deemed noisy and the final bodily representation is made of both interoceptive and exteroceptive information. In (**B**), the interoceptive information is incongruent with the exteroceptive ones (high heart rate when seeing a rabbit). As a consequence, the interoceptive stream will be deemed as noisy and the final bodily representation will be constructed using exteroceptive information only.

We carried out the simulation with values of ${\alpha }_{d}$ going from 1 to 50, representing the first 50 DPD episodes, and adapted the values of observations and inferred hidden states post-hoc to reflect realistic heart rates (used here as an example of interoceptive information stream). The results (Fig. 3a) show how when heart rate (observations) increases above a certain threshold its inferred value (hidden states) stops reflecting it and drops to a normal, safety-signalling level. Heart rate is effectively cut off from all higher-level processing, as its inferred value is almost solely determined by top-down predictions. If we generalise this for a larger number (possibly all) of bodily sensory channels, we have a situation in which the body itself is cut off from high-level cognition, and, we argue, conscious experience, generating DPD symptoms. The simulations also show how the development of dissociation habits lowers the threshold heart rate values for triggering a DPD episode. That is, during early episodes a higher heart rate is needed to initiate a dissociative episode, whereas following recurring dissociative episodes, a much lower heart rate threshold is sufficient to trigger one (Fig. 3c). Finally, the simulated episodes also differ in duration (Fig. 3b), with dissociation lasting longer and longer as the number of past episodes increased, mirroring the typical course of the disorder (with chronic patients experiencing longer-lasting episodes^69^). We illustrate the difference between congruent and incongruent interoceptive and exteroceptive information streams in Fig. 4.

## Discussion

The aim of the current paper was to provide a model of depersonalization as a downregulation of interoceptive prediction errors (here referred to as interoceptive silencing), which gives rise to a disembodied self. We simulated the behaviour of an agent subjected to a situation of high psychophysiological activation perceived as inescapable. Specifically, the agent was exposed to high levels of interoceptive signalling (conceptualised as increased heart rate in the figures for illustrative purposes) that could potentially be explained away as the presence of an imminent threat. However, the heightened interoceptive signal stream was accompanied by exteroceptive signalling that can be meaningfully resolved as the absence of a sensorially perceivable threat. These two “incoherent” feedforward streams would generate a dramatic increase in prediction errors. In our simulation, the artificial agent effectively silenced bottom-up interoceptive prediction errors, whilst exteroceptive ones remained unaffected. This interoceptive silencing would lead to a scenario in which the body is not anymore the physical medium through which the outside world is experienced. Consequently, the “transparency” that characterises the phenomenological experience of being a self is now lacking, and will lead to the phenomena of depersonalization. Finally, repeated exposure to similar situations led our agent to be more inclined to experience a depersonalization episode even in the face of a less triggering situation. That is, our agent was increasingly more disengaged from bodily responses and even relatively innocuous interoceptive stimuli triggered a dissociative response, which ends lasting increasingly longer.

It is important to note that the mathematical model used for our simulation is a very simplified version of a possible real-world scenario, and it is not intended to fully capture the complexity of DPD, but just to illustrate a possible dissociation mechanism under the PC and Active Inference frameworks. Our starting point (exteroceptive observations signalling safety and interoceptive ones signalling danger) is itself a simplification. In reality, the interoceptive activation must have a trigger, which would itself be part of the observations. The physiological activation would therefore be a preparatory response to a predicted threat that the individual has learned to associate with danger. One example of how the connection between trigger and physiological arousal might originate is that of child abuse. The child would likely develop an automatic physiological response to the dangerous adult, with physical contact being a possible trigger. Experiencing violence from a caregiver is easy to perceive as inescapable, as that same caregiver is responsible (and indispensable) for the survival of the child (as they provide food, shelter, etc.), and fighting back or fleeing would very likely jeopardise this. On the other hand, the need of the child (or, to use the same language as above, their preference) to have a reliable and caring caregiver they can depend on is incompatible with the hurt they cause them. They are thus left with dissociation as their only option to cope with the situation. Crucially, as the physiological response itself is unaffected by dissociation, this would keep arising in presence of the aforementioned trigger, in this example physical contact, even if this presents itself in a harmless situation. This brings us back to our starting point, with a high physiological activation signalling the presence of danger in form of a harmless external observation (e.g., a gentle caress from a loving partner). There is then the issue of the perceived inescapability, which we assume for simplicity in the simulation. As mentioned, suffering abuse from a caregiver is very likely to be perceived as inescapable, but this does not necessarily translate to all successive experiences of physical contact. The perception of inescapability would therefore have to be itself learned in association with the trigger, causing the individual to (incorrectly) generalise their inability to fight back/flee to all situations in which they are touched.

Of course, this is just an example, but quite a relevant one, as it has been shown that many individuals with DPD have a history of childhood trauma^65,66^. However, the same situation might be reached through different avenues, the exploration of which goes beyond the scope of this paper.

As mentioned above, we suggest that the interoceptive silencing mechanism takes place at the AI level. Indeed, while the posterior insula is thought to integrate multimodal sensory information giving rise to an implicit and “in the moment” body-awareness, sensory information processing in the AI culminates in a more explicit, narrative, and “affectively coloured” body-awareness. The phenomenological consequence of such a suppressive mechanism would be that of a depersonalisation feeling that, despite being perceived as potentially belonging to one's own body in the present moment, does not fit in the more extended and historically coherent selfhood. This would also explain the non-delusional character of depersonalisation experiences; given that sensory processing at the posterior insula level is still intact, the individual understands that such feeling of detachment from its own body is not real despite its apparent vividness and therefore perceived as “akin to a dream”.

We propose that the process of exteroceptive and interoceptive information integration happens in the insula, one of the key neural regions involved in DPD. Looking at Eq. (27), both terms can be read as a collection of prediction errors weighted by their variance (i.e. inverse precision), which in neurobiological models of predictive coding correspond to error neurons (whose activity represent variance-weighted prediction errors) with recurrent inhibitory connection (whose synaptic strength represents the variance associated with a particular prediction error^56,59^). We suggest the interoceptive error neurons represented by the first term of the equation (signalling the discrepancy between interoceptive observations and prediction coming from higher sensory areas) are located in the anterior insula. Importantly, these error neurons are weighted not only by their variance, but also by the probabilities associated with different policies (**c**), and by the effect of those policies (**θ**). When dissociation occurs, interoceptive prediction errors are effectively inhibited by the effect of this policy (as **θ** increases for interoceptive information channels). In the brain, this variance (or precision) regulation could happen through modulatory connections from the representation neurons in the prefrontal cortex to the (interoceptive) error neurons in the AI. Mathematically this would impact error neurons’ activity with an additive (or subtractive, depending on whether these are excitatory or inhibitory connections) effect, but for the sake of mathematical simplicity and synthesis we made the effect multiplicative in our model.

This process of sensorial multimodal prediction error explanation would culminate in a unified representation of the bodily self^70,71^. Recent studies in rodents provided strong evidence in support of the role of the posterior insula in integrating interoceptive and exteroceptive information^72^ and in the role of the AI in computing an ongoing interoceptive representation used to predict future interaction between interoceptive and exteroceptive states^73^ (for a discussion see^74^). Our hypothesis is also in line with the evidence that disorder of the self may arise as a consequence of a structural disconnection between the insular cortex and higher order frontal structures^75–77^.

Our model represents a theoretical and computational advance of previous conceptualisations (e.g.,^63^) within an active inference framework. In line with Gatus and colleagues^10^ we propose that DPD arises as a consequence of the suppression of interoceptive signals (deemed unreliable) whilst other sensory modalities remain intact. As illustrated by our simulation, to account for the prediction errors generated by incoherent streams of information, multisensory integration processes rely on sensory input from modalities other than interoception (e.g., exteroception, proprioception). Another recent account, put forward by Ciaunica and colleagues^67^, hypothesised that DPD may be the result of the “overthinking” of processes that would otherwise happen in the background (e.g., without being the focus of attention). The authors suggest that attenuation of the self is crucial to an intact sense of agency, and that therefore such overthinking would lead to an excessive exertion of control over one’s own actions and perceptions (loss of transparency). This would ultimately produce a split in the sense of self, whereby individuals with DPD would present with a dissociation “between the ‘I’ as a subject of an experience and the ‘me’ as an object of my awareness” (^67^, p.8). Whilst an extensive discussion of this rich and intriguing model goes beyond the scope of the current paper, we feel the two models are compatible, perhaps each offering insights into different stages of the dissociative process. While the ‘overthinking’ account may be a source of dissociation in self-awareness as suggested by Ciaunica and colleagues, another possibility is that developmentally this overthinking is itself caused by the kind of conflictual situations predicted by our model, leading individuals to attempt to ‘think away’ the interoceptive predictions they cannot more automatically explain away based on exteroceptive, or more integrated predictions about the source of felt arousal, as in the abuse examples suggested above.

At the neurobiological level, increased availability of both glutamate and serotonin has been linked to DPD. Use of NMDA receptor agonists, such as cannabinoids or ketamine, has been shown to induce depersonalization episodes or even chronic depersonalization disorder^8,9^. Similarly, recreational use of hallucinogens, such as s lysergide (LSD), psilocybin and dimethyltryptamine (DMT), as well as 3,4-Methyl​enedioxy​methamphetamine (MDMA), have been associated with transient and chronic DPD^78^. Depersonalization may hence represent a response to an excessive emotional experience induced by increased activity of glutamate and serotoninergic pathways. Speculatively, the interoceptive silencing we propose is at the core of depersonalization may represent an attempt to counteract such an intense experience, especially when repeated over time. This explanation is also in line with the evidence showing that DPD is associated with hypo, rather than hyper, autonomic activity, suggesting a selective inhibition of emotional processing^22,79^.

Clinical manifestations of interoceptive disruption also provide support to our conceptualisation. DPD patients showed altered neurophysiological^80^ and cardiac^81^ cortical and brainstem representation, suggesting difficulties in processing interoceptive signals. Similarly, individuals with functional neurological disorder show higher levels of dissociative behaviours when compared to controls, as well as lower accuracy during interoceptive tasks^82^. Additionally, compromised interoceptive accuracy with concomitant high interoceptive sensibility has been observed in individuals with functional seizures, often arising from dissociative states^83^. As put forward by Palser and colleagues^84^, individuals with high trait interoceptive sensibility may be more susceptible to anxiety when they fail to correctly attribute interoceptive signals to emotional states. As such, individuals with this profile may be more prone to developing DPD symptoms.

Our hypothesis is also in line with behavioural data. In a somatosensory paradigm, investigating whether subjects with low and high DPD traits differentially process information related to self (i.e. viewing touch being delivered on one’s own face) versus information related to someone else (i.e. viewing touch being delivered on someone else’s face), the authors found no difference between self and other processing in the high-trait DPD group^85^. This impairment in self-other distinction observed in high-trait DPD individuals may be linked to an inability to differentiate between signals arising from one’s own body (e.g., interoceptive) and signals observed on someone else’s body (e.g., exteroceptive). Hence, rather than processing signals related to the self as the subject of experience, DPD patients may tend to attribute the cause of all sensory information to external sources.

## Conclusions

We presented a theoretical model that explains DPD under the predictive coding and active inference frameworks. In our model, the depersonalisation phenomena arise from the downregulation of interoceptive prediction errors (interoceptive silencing). To illustrate this, we simulated the behaviour of an agent exposed to conflictual information coming from two different information streams: the interoceptive information stream, signalling the presence of an imminent threat, and exteroceptive ones suggesting the absence of such alleged threat. By updating its policies, the agent will downregulate the incoming interoceptive prediction errors, computing a bodily self that relies on exteroceptive information only. This process will give rise to a disembodied self and therefore, to the phenomenology akin to depersonalization. When repeated, this aberrant process of interoceptive silencing will cause a habit update, thus triggering depersonalisation episodes more frequently and in spite of less triggering situations. This model represents a step forward in the understanding and characterization of DPD, which could open new avenues for treatment. For instance, manipulation of multisensory paradigms including exteroceptive and interoceptive components could be used in rehabilitative settings to restore balanced multisensory integration processes (see^86,87^ for an example). Similarly, repeated focused exposure to interoceptive tasks (such as experience sampling methods or ecological interoceptive tasks; see^88,89^ for an example) may attenuate interoceptive silencing over time.

## Methods

We developed a simulated agent with $N$ sensory channels from which it received information about the outside world (exteroceptive channels) and from its own organism (interoceptive channels). During the simulation it was presented with a series of observations. By inverting its generative model, the agent inferred the value of lower-level continuous hidden states (one per sensory channel) and the integrated higher-level discrete hidden state (obtained by integrating information about all lower-level hidden states), as well as the best policy given sensory evidence, habits and preferences. After picking a policy, it updated its habits accordingly. In this section we describe the generative model and give an overview of the Active Inference algorithm.

### Generative model

In our agent’s generative model, the joint probability of observations $\bf o$ and lower-level hidden states $\bf s$ at the time point $t$ is:

1 $$p({\mathbf{o}}_{t},{\mathbf{s}}_{t}\mid {\mathbf{o}}_{1:t-1},{\mathbf{s}}_{1:t-1},{\varvec{\upphi}})$$

with ${\varvec{\upphi}}$ being a vector specifying the agent’s preference (expressed as a probability) about any of $K$ higher-level hidden states being active (see below). For simplicity, we assume that the agent believes observations and states not to spontaneously (i.e., in absence of actions) change over time, eliminating time dependency:

2 $$p({\mathbf{o}}_{t}\mid {\mathbf{o}}_{1:t-1},{\mathbf{s}}_{1:t},{\varvec{\upphi}})=p({\mathbf{o}}_{t}\mid {\mathbf{s}}_{t},{\varvec{\upphi}})$$

3 $$p({\mathbf{s}}_{t}\mid {\mathbf{s}}_{1:t-1},{\varvec{\upphi}})=p({\mathbf{s}}_{t}\mid {\varvec{\upphi}})$$

in which we have factorised

4 $$p({\mathbf{o}}_{t},{\mathbf{s}}_{t})=p({\mathbf{o}}_{t}\mid {\mathbf{s}}_{t},{\varvec{\upphi}})p({\mathbf{s}}_{t}\mid {\varvec{\upphi}})$$

dropping temporal indexing

5 $$p(\mathbf{o},\mathbf{s})=p(\mathbf{o}\mid \mathbf{s},{\varvec{\upphi}})p(\mathbf{s}\mid {\varvec{\upphi}})$$

It is important to stress that here ${\varvec{\upphi}}$ does not reflect a belief, but preferences of the agent about the higher-level state it would rather be in (e.g., “safe” versus “in danger”, “full” versus “starving”, etc.), and we treat it as a categorical distribution representing mixing coefficients of a mixture of Gaussians. Following standard practice^54^ we then introduce a new binary variable ${\varvec{z}}$ with K elements, whose values must satisfy ${z}_{k}\in \{\mathrm{0,1}\}$ and ${\sum }_{k=1}^{K}{z}_{k}=1$. Its probability distribution is specified as:

6 $$p({\varvec{z}}\mid {\varvec{\upphi}})=\prod \limits_{k=1}^{K}{\phi }_{k}^{{z}_{k}}$$

Inferring the values of ${\varvec{z}}$ is equivalent to inferring what integrated state the agent finds itself in. Both observations $\bf o$ and lower hidden states $\bf s$ are continuous variables with Gaussian likelihoods. For simplicity we are having our agent assume that information channels are independent from each other and expect the value of each ${o}_{n}$ to be centred around ${s}_{n}$ with Gaussian noise, so that

7 $$p(\mathbf{s}\mid \mathbf{z})=\prod \limits_{k=1}^{K} \prod \limits_{n=1}^{N}N({s}_{n}\mid {\mu }_{k,n},{\sigma }_{k,n}^{(s)2}{)}^{{z}_{k}}$$

8 $$p(\mathbf{o}\mid \mathbf{s})=\prod \limits_{n=1}^{N}N({o}_{n}\mid {s}_{n},{\sigma }_{n}^{(o)2})$$

In our model policies are treated as hidden states, and the agent performs inference on them to select which one to enact. As before, we introduce a new binary variable $\mathbf{c}$ with M elements, whose values must satisfy ${c}_{m}\in \{\mathrm{0,1}\}$ and ${\sum }_{m=1}^{m}{c}_{m}=1$ and its probability distribution is specified as:

9 $$p(\mathbf{c})=\prod \limits_{m=1}^{M}{\pi }_{m}^{{c}_{m}}$$

where ${\pi }_{m}$ represents the prior probability of enacting a policy $m$. We allow that the agent changes its beliefs about ${\varvec{\uppi}}$, but not about ${\varvec{\upphi}}$, as the former represents habits, and the latter natural preferences to avoid some situations and seek others. Therefore, we place a Dirichlet prior on ${\varvec{\uppi}}$ only:

10 $$p({\varvec{\uppi}})=Dir({\varvec{\uppi}}\mid {\varvec{\upalpha}})$$

**Figure 5:**
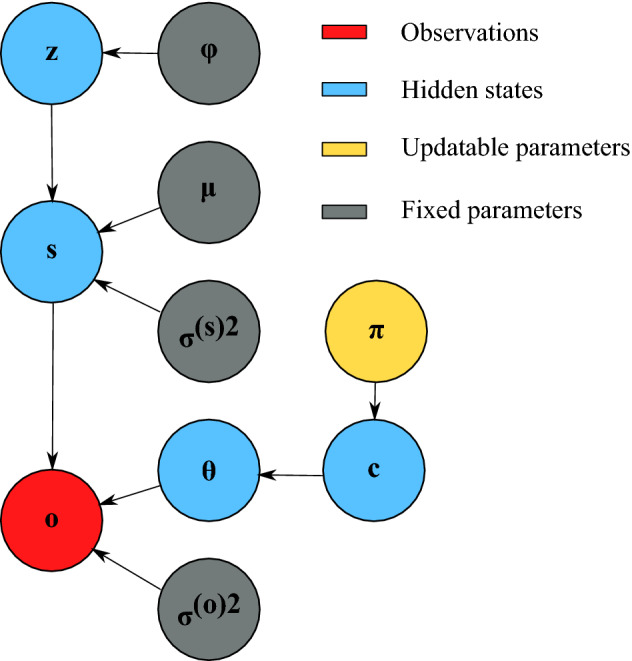
Graphical representation of causal dependencies in the generative model. Arrow direction specifies the directionality of the causal relationship.

The scenario we are trying to capture with our simulations is that of an abnormal physiological activation in the apparent absence of a threat, resulting in an incongruence between interoceptive and exteroceptive information. Normal reactions as “fight” or “flight” would bear no effect, as there would be nothing to fight or run away from. Thus, the only policy with an effect is dissociation, formalised as an increase of the variance in the likelihood mapping from interoceptive hidden states and interoceptive observations, allowing top-down prediction signals to dominate over sensory evidence (‘interoceptive silencing’). To avoid unnecessary complexity, we modelled the agent to have certain knowledge about the effects of policies (although in a biological agent this knowledge would be implicit, or unconscious). It is important to point out that this assumption is formalised in the structure of the generative model, and not by fixing the model’s parameters. Furthermore, it is worth noting that a possible, parallel effective policy during an episode of abnormal physiological activation (such as a panic attack) is arguably to seek help, that is, to sample social information (be it tactile, visual or auditory; and be it in adult^90^ or developmental timescale^91^). The alternative, regular availability of this course of action, e.g., social support or psychotherapy, might very well be a crucial element in preventing the development of depersonalisation disorder, or treating it, but this issue escapes the scope of the present model; here we just assume this social option is not available. In our model depersonalisation episodes take place only when the situation is perceived as close to inescapable, at least in the early stages of the disease (i.e., before dissociation habits develop). Thus

11 $$p(\mathbf{o}\mid \mathbf{s},\mathbf{c})=\prod \limits_{m=1}^{M} \prod \limits_{n=1}^{N}N({o}_{n}\mid {s}_{n},{\theta }_{m,n}{\sigma }_{n}^{(o)2}{)}^{{c}_{m}}$$

with ${\theta }_{m,n}>1 \forall m\in \mathbf{d}\wedge \forall n\in \mathbf{i}$ for dissociation policies in interoceptive channels ($\mathbf{i}$ representing interoceptive sensory streams and $\mathbf{d}$ dissociation policies) and ${\theta }_{m,n}=1 \forall m\notin \mathbf{d}\vee \forall n\notin \mathbf{i}$ for all other policies and channels. The joint (see Fig. 5 for the graphical model) thus becomes:

12 $$\begin{aligned} p(\mathbf{o},\mathbf{s},\mathbf{z},\mathbf{c},{\varvec{\uppi}}) &= p(\mathbf{o}\mid \mathbf{s},\mathbf{z},{\varvec{\upphi}},\mathbf{c},{\varvec{\uppi}})p(\mathbf{s}\mid \mathbf{z},{\varvec{\upphi}})p(\mathbf{z}\mid {\varvec{\upphi}})p(\mathbf{c}\mid {\varvec{\uppi}})p({\varvec{\uppi}}) \\ & = Dir({\varvec{\uppi}}\mid {\varvec{\upalpha}}) \left(\prod \limits_{m=1}^{M}{\pi }_{m}^{{c}_{m}} \prod \limits _{n=1}^{N}N({o}_{n}\mid {s}_{n},{\theta }_{m,n}{\sigma }_{n}^{(o)2}{)}^{{c}_{m}}\right) \left(\prod \limits_{k=1}^{K}{\phi }_{k}^{{z}_{k}}{\prod }_{n=1}^{N}N({s}_{n}\mid {\mu }_{k,n},{\sigma }_{k,n}^{(s)2}{)}^{{z}_{k}}\right) \end{aligned}$$

### Active inference

To make inferences about hidden states, the agent maximises Variational Free Energy (VFE):

13 $$VFE={E}_{q(\mathbf{s},\mathbf{z},\mathbf{c},{\varvec{\uppi}})}\left[ln\frac{p(\mathbf{o},\mathbf{s},\mathbf{z},\mathbf{c},{\varvec{\uppi}})}{q(\mathbf{s},\mathbf{z},\mathbf{c},{\varvec{\uppi}})}\right]$$

with $q(\cdot )$ being the approximate posteriors. Note that we are treating policies as hidden states, with the result of using a common algorithm for inference and action selection. This can be optimised by iteratively evaluating optimal solutions ${q}^{*}(\mathbf{z})$, ${q}^{*}(\mathbf{c})$, ${q}^{*}({\varvec{\uppi}})$ and ${q}^{*}(\mathbf{s})$ until convergence through an Expectation Maximisation (EM) loop. This entails the alternation of an *E step* in which the artificial agent estimates the values of $\widetilde{\mathbf{z}}=E\left[\mathbf{z}\right]$ and $\widetilde{\mathbf{c}}=E\left[\mathbf{c}\right]$, which will then be used for the subsequent *M step* for estimating optimal values for $\mathbf{s}$ and ${\varvec{\uppi}}$.

#### E step

In this step the agent determines which integrated hidden state is more likely to be active and which action to take. Optimal solutions can be found^54^ by evaluating

14 $$ln{q}^{*}(\mathbf{z})={E}_{q(\mathbf{s},\mathbf{c},{\varvec{\uppi}})}\left[ln\frac{p(\mathbf{o},\mathbf{s},\mathbf{z},\mathbf{c},{\varvec{\uppi}})}{q(\mathbf{s},\mathbf{z},\mathbf{c},{\varvec{\uppi}})}\right]$$

and

15 $$ln{q}^{*}(\mathbf{c})={E}_{q(\mathbf{s},\mathbf{z},{\varvec{\uppi}})}\left[ln\frac{p(\mathbf{o},\mathbf{s},\mathbf{z},\mathbf{c},{\varvec{\uppi}})}{q(\mathbf{s},\mathbf{z},\mathbf{c},{\varvec{\uppi}})}\right]$$

from which it can be shown that

16 $${\widetilde{z}}_{k}=\frac{{\rho }_{k}}{{\sum }_{j=1}^{K}{\rho }_{j}}$$

with

17 $$ln{\rho }_{k}=ln{\phi }_{k}-\sum \limits_{n+1}^{N}\frac{({\widetilde{s}}_{n}-{\mu }_{k,n}{)}^{2}+{\widetilde{\sigma }}_{n}^{2}}{2{\sigma }_{k,n}^{(s)2}}$$

and

18 $${\widetilde{c}}_{m}=\frac{{\rho }_{m}}{{\sum }_{v=1}^{M}{\rho }_{v}}$$

with

19 $$ln{\rho }_{m}=\psi ({\widetilde{\alpha }}_{m})-\psi \left({\sum \limits _{v=1}^{M}}{\widetilde{\alpha }}_{v}\right)- \sum \limits_{n+1}^{N}\left\{\frac{1}{2}ln({\theta }_{m,n}{\sigma }_{n}^{(o)2})+\frac{({o}_{n}-{\widetilde{s}}_{n}{)}^{2}+{\widetilde{\sigma }}_{n}^{2}}{2{\theta }_{m,n}{\sigma }_{n}^{(o)2}}\right\}$$

with ${\widetilde{z}}_{k}$ and ${\widetilde{c}}_{m}$ representing the estimated probabilities of integrated hidden state $k$ being active and of policy $m$ being enacted, respectively. Here ${\widetilde{s}}_{n}$ and ${\widetilde{\sigma }}_{n}^{2}$ are the mean and variance of ${q}^{*}({s}_{n})$, $\widetilde{{\varvec{\upalpha}}}$ are the updated parameters of ${q}^{*}({\varvec{\uppi}})$ (see M step) and $\psi (\cdot )$ is the digamma function. For the first iteration of the E step the model initialises these values to their prior:

20 $$\widetilde{\mathbf{s}}={{\varvec{\upmu}}}_{\gamma }$$

21 $$\widetilde{{\varvec{\upsigma}}}^{2}={{\varvec{\upsigma}}}^{(o)2}$$

and

22 $$\widetilde{{\varvec{\upalpha}}}={\varvec{\upalpha}}$$

with $\gamma$ being the index of the preferred higher-level hidden state.

#### M step

The estimated values of $\widetilde{\mathbf{z}}$ and $\widetilde{\mathbf{c}}$ can now be used to evaluate

23 $$ln{q}^{*}({\varvec{\uppi}})={E}_{q(\mathbf{s},\mathbf{z},\mathbf{c})}\left[ln\frac{p(\mathbf{o},\mathbf{s},\mathbf{z},\mathbf{c},{\varvec{\uppi}})}{q(\mathbf{s},\mathbf{z},\mathbf{c},{\varvec{\uppi}})}\right]$$

and

24 $$ln{q}^{*}(\mathbf{s})={E}_{q(\mathbf{z},\mathbf{c},{\varvec{\uppi}})}\left[ln\frac{p(\mathbf{o},\mathbf{s},\mathbf{z},\mathbf{c},{\varvec{\uppi}})}{q(\mathbf{s},\mathbf{z},\mathbf{c},{\varvec{\uppi}})}\right]$$

from which the agent can straightforwardly update

25 $${\widetilde{\alpha }}_{m}={\alpha }_{m}+{\widetilde{c}}_{m}$$

where ${\widetilde{\alpha }}_{m}$ is the approximate posterior value of ${\alpha }_{m}$. On the other hand, estimating the optimal posterior values of hidden states $\widetilde{s}_n=E[s_n]$ requires the deployment of a gradient ascent loop, in which the values of $\widetilde{s}_n$ are iteratively evaluated until convergence. Here we use the Newton’s method, so

26 $${\widetilde{s}}_{n}\leftarrow {\widetilde{s}}_{n}-\frac{\frac{\partial {q}^{*}({s}_{n})}{\partial {s}_{n}}}{\frac{{\partial }^{2}{q}^{*}({s}_{n})}{\partial {s}_{n}^{2}}}$$

with

27 $$\frac{\partial {q}^{*}({s}_{n})}{\partial {s}_{n}}= \sum \limits _{m=1}^{M}{\widetilde{c}}_{m}\frac{({o}_{n}-{s}_{n})}{{\theta }_{m,n}{\sigma }_{n}^{(o)2}}-\sum \limits _{k=1}^{K}{\widetilde{z}}_{k}\frac{({s}_{n}-{\mu }_{k,n})}{{\sigma }_{k,n}^{(s)2}}$$

and

28 $$\frac{{\partial }^{2}{q}^{*}({s}_{n})}{\partial {s}_{n}^{2}}=-\sum \limits_{m=1}^{M}\frac{{\widetilde{c}}_{m}}{{\theta }_{m,n}{\sigma }_{n}^{(o)2}}-\sum \limits_{k=1}^{K}\frac{{\widetilde{z}}_{k}}{{\sigma }_{k,n}^{(s)2}}$$

For evaluating ${\widetilde{\sigma }}_{n}$ we make use of the Laplace approximation, so the precision (inverse variance) is given by

29 $${\widetilde{{\uptau}}}_{n}=-\frac{{\partial }^{2}{q}^{*}({s}_{n})}{\partial {s}_{n}^{2}}$$

and

30 $${\widetilde{{\upsigma}}}_{n}=\frac{1}{{\widetilde{\tau }}_{n}}$$

These values are then used to re-evaluate $\widetilde{\mathbf{c}}$ and $\widetilde{\mathbf{z}}$ in the next E step.

#### Habits update

The EM algorithm is repeated until all the inferred values $\widetilde{\mathbf{c}}$, $\widetilde{\mathbf{z}}$, $\widetilde{{\varvec{\upalpha}}}$ and $\widetilde{\mathbf{s}}$ (all rounded to 6 decimal places) converge. Of these, only $\widetilde{{\varvec{\upalpha}}}$ is used for updates, as the others represent contingent states. Thus:

31 $${\varvec{\upalpha}}\leftarrow \widetilde{{\varvec{\upalpha}}}$$

representing habits update.

### Simulation

For our simulation, we set (arbitrarily)

$$N=10$$

$$\mathbf{i}=1{:}7$$

$$M=3$$

$${\varvec{\upalpha}}=\left[100, 100, 1\right]$$

$$d=3$$

$${\sigma }_{n}^{(o)2}=25 \, \forall n \, \in \mathbf{n}$$

$${\sigma }_{k,n}^{(s)2}=100 \, \forall k \, \in K\wedge \forall n\in \mathbf{n}$$

$$K=2$$

$${\mu }_{1,n}=20 \, \forall n \, \in \mathbf{n}$$

$${\mu }_{2,n}=80 \, \forall n \, \in \mathbf{n}$$

$${\phi }_{1}=0.99$$

$${\phi }_{2}=0.01$$

$${o}_{n}=80 \, \forall n \, \in \mathbf{i}$$

$${o}_{n}=20 \, \forall n \, \notin \mathbf{i}$$

$${\theta }_{d,n}=100 \, \forall n \, \in \mathbf{n}$$

$${\theta }_{m,n}=1 \, \forall n \, \in \mathbf{n}\wedge \forall m\notin \mathbf{m}$$

where $\mathbf{n}$ and $\mathbf{m}$ are vectors containing all channel (from $1$ to $N$) and policy (from $1$ to $M$) indexes, respectively. This means that our simulated agent had 10 sensory channels, 7 of which interoceptive and 3 of which exteroceptive. It had 3 available policies, but it was much more prone to enact 2 of them (the non-dissociative ones). It could find itself in 2 possible higher-level states, the first of which (“safety”) it strongly favoured over the other (“danger”). These two states were associated with low (20) and high (80) mean values of lower-level hidden states, respectively. If the dissociation policy $d$ were enacted, the agent would increase the variance of the lower-lever interoceptive states ${\mathbf{s}}_{\mathbf{i}}$. We initialised all $\mathbf{s}$ to 20 at the start of every simulation, reflecting a starting point of relative tranquillity before the onset of the physiological over-activation. We carried out two simulations: the first one illustrating habits formation, exactly as described above, and the second one simulating an actual depersonalization episode, with the agent exposed to a changing set of observations (exteroceptive observation fixed to $20$ and interoceptive ones starting from $20$, quickly rising to $80$, plateauing and then slowly decaying back to $20$). In the latter, after the first time-point, ${\widetilde{{\varvec{\upsigma}}}}$ and $\widetilde{\mathbf{s}}$ are initialised as those to which the algorithm converged at the previous time point, as opposed to prior values. We did not need to sample actions, as interestingly the estimated values of $\widetilde{\mathbf{c}}$ were always either very close to $0$ or very close to $1$ (see “Results” section).

## Data Availability

No datasets were generated or analysed during the current study.
